# Effect of Low Pressure on Surface Roughness and Morphological Defects of 4H-SiC Epitaxial Layers

**DOI:** 10.3390/ma9090743

**Published:** 2016-08-31

**Authors:** Jichao Hu, Renxu Jia, Bin Xin, Bo Peng, Yuehu Wang, Yuming Zhang

**Affiliations:** Key Laboratory of Wide Band-Gap Semiconductor Materials and Devices, School of Microelectronics, Xidian University, 2 South Taibai Road, Xi’an 710071, China; hujichao0613@163.com (J.H.); xinbin280@163.com (B.X.); illidan2008007@163.com (B.P.); wangyh@mail.xidian.edu.cn (Y.W.); zhangym@xidian.edu.cn (Y.Z.)

**Keywords:** low pressure growth, 4H-SiC homoepitaxy, surface diffusion length, minimization mode of surface energy

## Abstract

In this work, 4H-SiC epilayers are performed on 4° off-axis substrates under low pressure condition by horizontal hot wall chemical vapor deposition (HWCVD) with a standard chemistry of silane-propane-hydrogen, which focuses on the effects of growth pressure on morphology, basal plane dislocations (BPDs) and crystalline quality. It is found that morphological defects reduce with the decreasing of growth pressure, since the surface diffusion length of absorbed adatoms increases under low growth pressure, which suppresses the nucleation of adatoms on terraces and the formation of morphological defects. However, as the surface diffusion length increases under low growth pressure, the difference of growth velocity at steps is enhanced, which leads to the extension of the steps’ width and the formation of step-bunching. Besides variation of surface diffusion length, the phenomenon described above can be correlated with different dominate modes for the minimization of surface energy at varied growth pressure. Because of the contrary influence of increased C/Si ratio and enhanced step-flow growth on the propagation of BPDs, the dislocation densities of BPDs and threading edge dislocations (TEDs) in epilayers grown at varied pressures remain basically unchanged. The crystalline quality is almost independent of growth pressure based on high resolution X-ray diffraction (HRXRD) measurements.

## 1. Introduction

Because of higher power conversion efficiency, 4H Silicon carbide (4H-SiC) devices have benefits in wind turbine power converters [1,2,3], photovoltaic inverters [4,5] and hybrid electric vehicles [6,7,8]. Among 4H-SiC devices, 4H-SiC metal-oxide-semiconductor field effect transistors (MOSFETs) and Schottky barrier diodes (SBDs) with voltage ratings of 1.2 kV and 1.7 kV are the most widely used in power applications. Homoepitaxial growth of 4H-SiC layers is a key step in the fabrication of 4H-SiC power devices since high-quality 4H-SiC epilayers with low defects density, low doping concentration and good morphology are necessary [9]. The most established technique for growth of epitaxial layers of SiC is chemical vapor deposition (CVD) using silane (SiH_4_), and propane (C_3_H_8_) as precursors. A significant topic is the defects in epilayers, which impede devices’ performance. Epitaxial layers grown at high growth rate on 4° off-axis substrates suffer from step-bunching and triangular defects (TDs) [10,11,12]. It is reported that step-bunching causes an increase in the leakage current of SBDs [13] and has a negative impact on the channel mobility or the oxide breakdown characteristics in MOSFETs [14]. Triangular defects are thought to be device killers [15,16]. The formation of triangular defects may be attributed to the larger terrace widths resulting in two-dimensional nucleation [17]. Leone et al. [10] have studied the effect of the growth temperature on the formation of step-bunching and trianular defects in standard process. They found that, with temperature increase, a reduction of defects was seen but a significant increase of step-bunching was observed. In the work by Dong et al. [18], the effect of Cl/Si ratio on triangular defects was investigated. They found that triangular defects can be effectively suppressed at high Cl/Si ratio condition. In our previous work, we preliminarily studied the impacts of growth pressure on surface roughness and morphological defects of 4H-SiC epitaxial layers [19]. Besides morphological defects, basal plane dislocations (BPDs) which lead to increasing the forward voltage drift in SiC bipolar devices [20] or the reverse leakage current in unipolar devices [21] is another problem for epitaxial layers performed on off-axis substrates. Because of considerably higher elastic energy of dislocation per unit growth length, BPDs in the substrate deflect to threading edge dislocations (TEDs), which are less harmful to SiC devices in the epilayer during SiC homoepitaxial growth [22]. The conversion of BPDs to TEDs is correlated with growth condition [23,24], substrate pretreatment [25,26], etc. Even though several groups have investigated formation of morphological and crystal defects on off-axis 4H-SiC substrates using CVD, no consensus has been reached, since the approaches and the parameters of processes are different in each of the cases. Moreover, there are few reports about the effect of the growth pressure on the formation of morphological defects and the conversion of BPDs to TEDs. Therefore, it is necessary to study how to increase growth rate and improve morphology and crystallographic defects of 4H-SiC epilayers grown on the substrates with low off-axis angle at low pressure.

In this paper, we investigate 4H-SiC epilayers grown on 4° off-axis substrates in horizontal CVD under low growth pressure (<100 mbar), which emphasizes on the impact of growth pressure on surface morphology and the correlations between the surface roughness and morphological defects. Based on analysis of present results, the effect of growth pressure on conversion of BPDs to TEDs is discussed.

## 2. Results and Discussion

### 2.1. Effect of Growth Pressure on Growth Rate

**Figure 1:**
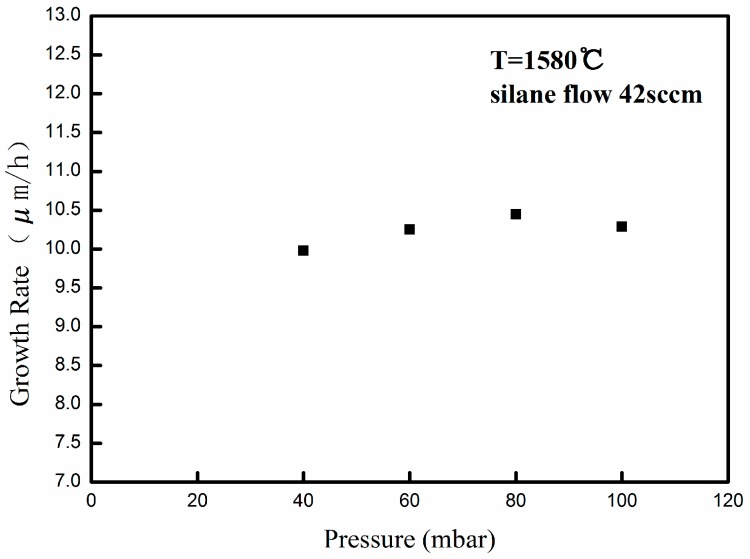
Dependence of 4H-SiC epitaxial growth rate on process pressure.

In order to systematically investigate the dependence of process pressure on the growth rate in low pressure range, the growth pressures were arranged between 40 and 100 mbar, and the other process parameters, such as silane flow rate (42 sccm), the process temperature (1580 °C) and C/Si ratio (1.0) are kept constants. As shown in Figure 1, the growth rate remains relatively constant between 40 and 100 mbar, approximately 10 μm/h, which indicates that the growth pressure has little impact on epilayer growth rate in low pressure range (<100 mbar). The relation between growth pressure and growth rate in lower pressure range is different from that in higher pressure range, for which the growth rate decreases rapidly as growth pressure increases in the 100–500 mbar range [27].

**Figure 2:**
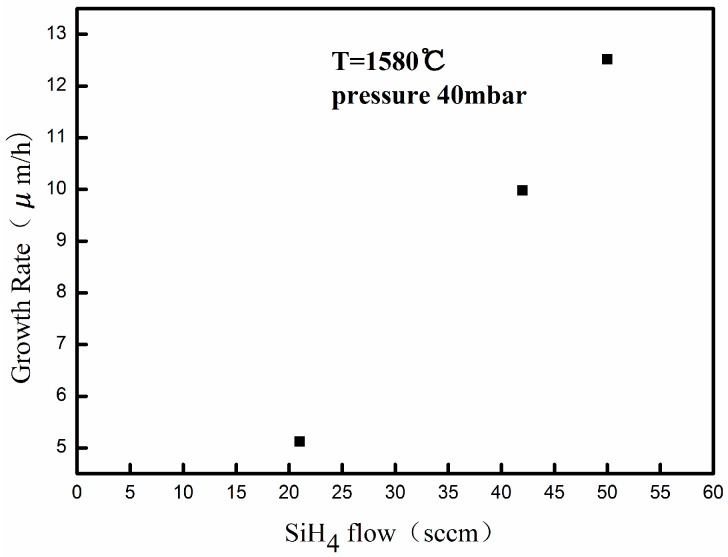
Growth rate of 4H-SiC epitaxial layers for various silane flow rate.

The impact of silane flow rate on the growth rate at low pressure was investigated. The silane flow rate varied between 21 and 50 sccm (the maximum silane flow rate of this CVD reactor). Figure 2 presents the results of the growth rate as a function of silane flow rate. The silane flow rate of 21 sccm produced a growth rate of 5 μm/h, while the maximum silane flow rate of 50 sccm produced a growth rate of approximately 13 μm/h, indicating a linear increase in growth rate with the silane flow rate. The epilayer surface grown with maximum silane flow rate of 50 sccm is specular with few morphological defects in optical microscope, which demonstrates that gas phase homogeneous nucleation that arises from supersaturation of silicon can be effectively suppressed at low growth pressure. It can be concluded that the growth rate will be elevated further with the increase of precursors flow rates to a certain extent at low pressure. Meanwhile, the surface morphology and structural quality of films grown at high growth rate under low pressure will not be degraded.

**Figure 3:**
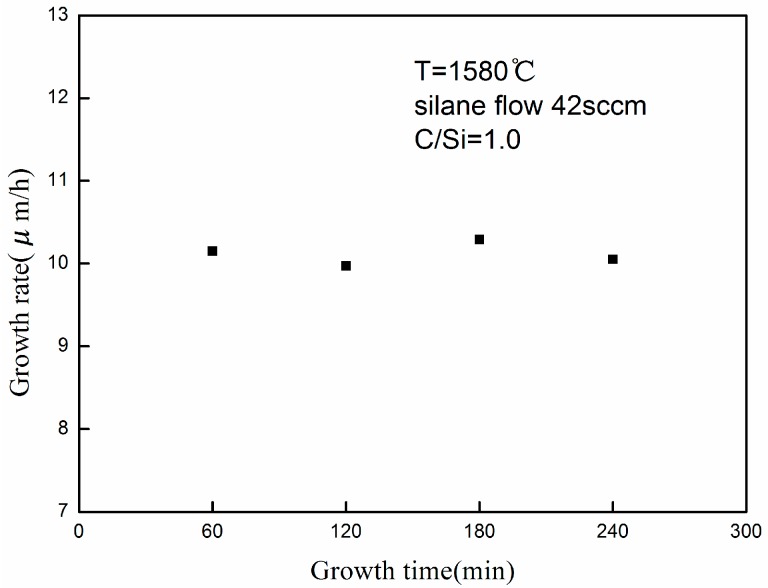
Growth rate of 4H-SiC epitaxial layers for various growth time.

To verify the stability and reproducibility of the thick epitaxial layers growth process, long growth runs were conducted to fabricate epitaxial films. The process parameters in this experimental section are the same as described above. Growth rates of 4H-SiC epitaxial layers for various growth time are shown in Figure 3. As the figure shows, the average growth rates remained constant (~10 μm/h) for various growth times. Films up to 50 μm were specular with occasional morphological defects, which indicates that the growth process at low pressure was of good stability and reproducibility.

### 2.2. Effect of Growth Pressure on Surface Roughness and Morphological Defects

**Figure 4:**
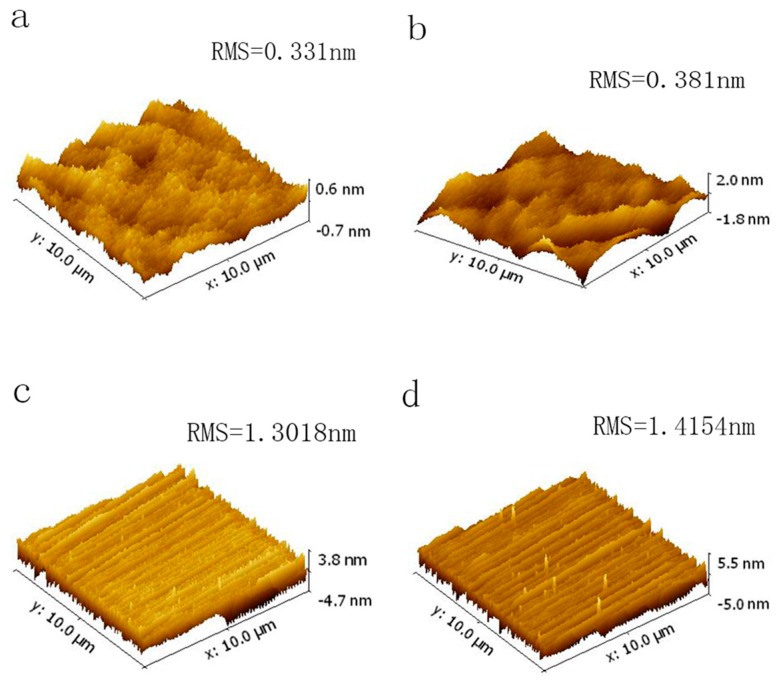
Atomic force microscopy (AFM) images (10 μm × 10 μm) of approximately 20-μm-thick 4H-SiC epitaxial layers grown under different pressures: (**a**) 100 mbar; (**b**) 80 mbar; (**c**) 60 mbar; (**d**) 40 mbar.

Surface roughness and morphological defects are key issues as the growth rate is effectively elevated. The impacts of process pressure on the surface roughness and morphological defects of epilayers are investigated in detail. The epilayers are grown on substrates cut from a wafer with homogenous surface condition and distributions of substrate defects under the same growth processes except for the growth pressure to avoid the impacts of other factors on surface roughness and morphological defects. All the epilayers have smooth and mirror-like surfaces viewed under an optical microscope. Figure 4 shows the atomic force microscopy (AFM) images of epilayers grown at different pressures: 40 mbar, 60 mbar, 80 mbar and 100 mbar. At growth pressure of 100 mbar, epilayer surface presents protuberances without step structures, and these protuberances are discontinuous as shown in Figure 4a. The roughness root mean square (RMS) of epilayers grown at 100 mbar is 0.331 nm. As the growth pressure reduced to 80 mbar, the protuberances elongate to discontinuous crossovers on the surface, indicating the anisotropy of the elongated protuberances (or steps) which caused by the difference in growth rates along the [10-10] and [11,12,13,14,15,16,17,18,19,20] directions [28] (Figure 4b). The RMS of epilayer grown at 80 mbar is 0.193 nm. Even though the epilayer exhibits a step-bunching free surface, it is reasonable to believe that 80 mbar is the initial stage of bunched steps. At the growth pressure of 60 mbar, the undulations are much more continuous across the surface (Figure 4c). Macrosteps perpendicular to the [11,12,13,14,15,16,17,18,19,20] direction appear on the surface, which causes the higher surface roughness (1.3018 nm). The width and height of macrosteps are 130–250 nm and 3–5 nm, respectively. At the growth pressure of 40 mbar, the width and height of macrosteps keep almost unchanged, adopting values of 150–300 nm and 3–5 nm, respectively. The RMS of epilayers grown at 40 mbar is 1.4154 nm. It is apparent that the variations of growth pressure influence surface roughness of the grown epilayers.

The increase of surface roughness may attribut to the following reasons: firstlyf, due to the difference in energy cost for deposition, there is a different step velocity between different basal planes of the stacking sequence [29]. It has been calculated that in 4H-SiC, the A and B planes are growing faster than the C plane [30,31]. In the early days, Kimoto et al. gave an expression of the growth rate of the step for chemical vapor deposition of 6H-SiC by the following equation [32]:

(1) $$\nu_{\mathrm{step}}=\frac{4J\lambda_{s}h_{0}}{n_{0}h}$$

where *J*, *n*_0_, *h*_0_, *h* and *λ*_s_ are the flux of reactants, the density of adatom sites, the spacing of the 6H-SiC {0001} face, the height of macrostep and surface diffusion length, respectively. This equation indicates that the growth rate of the step is proportional to the flux of reactants and surface diffusion length. Based on the stagnant layer model reported in references [33,34], mass transport from gas phase to wafer surface through the stagnant layer is increased under low pressure. To crystal growth, the surface diffusion length is a critical parameter since it determines whether crystal growth proceeds through step-flow or two-dimensional nucleation [32]. Increase of surfaces diffusion length at reduced growth pressure may be ascribed to the decreased supersaturation, which leads to the restraint of nucleation of adatoms on the terraces and reduction of nucleus density. Step velocity increases as the flux of reactants and surface diffusion length increase at low pressure. As a consequence, step-bunching may form due to the enhancement of difference of growth velocity at steps. On the other hand, it is demonstrated that the relative mole fraction of C-containing gaseous species increases with reduced growth pressure in a CVD reactor using a standard chemistry of silane-propane-hydrogen [35,36,37]. In other words, the effective C/Si ratio on the surface increases at lower pressure even though the inlet C/Si ratio is kept constant. It is reported that surface morphology is affected by the C/Si ratio [10,29,33,38]. Step-bouncing or surface roughness tend to enhance with increasing C/Si ratio, i.e., C-rich condition. High C/Si ratio may change surface migration of absorbed adatoms and incorporation probability at steps [32]. Besides, from a viewpoint of surface energy, the origin of step-bunching may be correlated with the surface equilibrium process in which the free energy is minimized during crystal growth [39,40,41]. According to the report of Kojima et al., the surface energy in Si-rich condition and in C-rich condition is different [26]. Therefore, variation tendency of step-bunching at varied growth pressure may be due to the difference of surface energy caused by variation of effective C/Si ratio.

**Figure 5:**
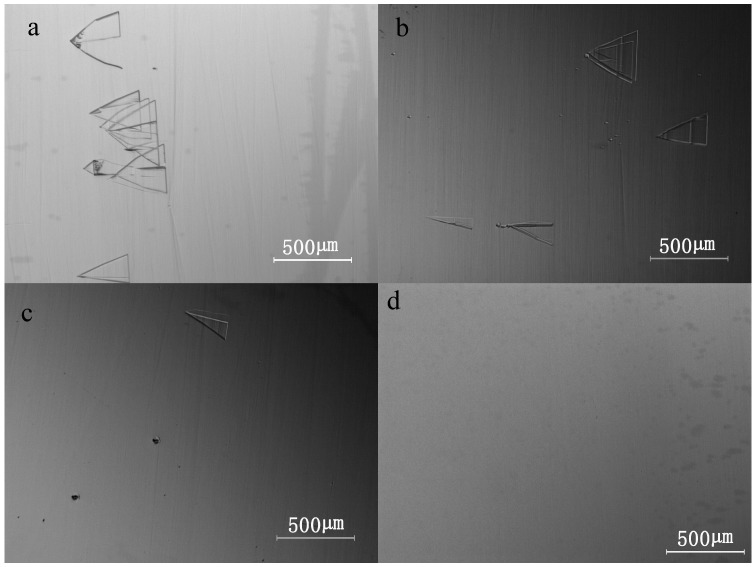
Optical microscopy images of approximately 20 μm thick 4H-SiC epitaxial layers grown under different pressures: (**a**) 100 mbar; (**b**) 80 mbar; (**c**) 60 mbar; (**d**) 40 mbar.

The optical microscope images of 20 μm thick samples performed at pressure varyingying between 40 and 100 mbar are shown in Figure 5. Downfall defects are observed on all samples. The densities of triangular defects for epitaxial layers grown at different pressures are shown in Table 1. It can be concluded that the amount of triangular defects reduces with the growth pressure decrease, which could be attributed to the increased surface diffusion length at lower pressure. With reduced growth pressure, surface migration of adsorbed species is enhanced, which means adsorbed species are more likely to reach the step and contribute to the step flow growth [32]. Therefore, 2D nucleation of adatoms on the terraces is suppressed. Meanwhile, the reduction of triangular defects shows that the step-flow growth is enhanced under lower pressure condition. It suggests that morphological defects tend to appear more frequently on the epilayer surfaces without step-bunching than on the surfaces with step-bunching. Similar results have been reported by Leone et al. [10] and Dong et al. [18] in studies with other growth processes and parameters. Apart from surface diffusion length, the phenomenon observed above relates to the minimization of surface energy at varied pressures. Besides step-bunching, surface energy can also be minimized by the formation of morphological defects. At lower pressure, the surface energy is mainly released by step-bunching. In contrast, the formation of morphological defects is the dominating mode in the minimization of surface energy at relatively higher pressure.

### 2.3. Effect of Growth Pressure on Crystal Defects and Crystal Quality

**Figure 6:**
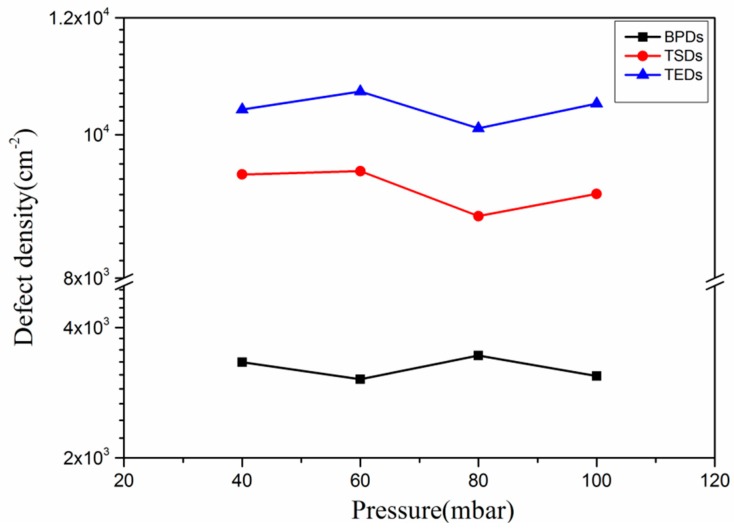
Dislocation densities of epitaxial layers grown at different pressures.

According to the discussion in Section 2.2, the effective C/Si ratio on the surface is increased at lower pressure. Ohno et al. [24] reported that higher C/Si ratios could promote the conversion of BPDs to TEDs. Therefore, we speculated that, previously, the BPDs density decreased with the reduction of growth pressure. However, it seems that the dislocation densities of epilayers are not influenced by growth pressure as shown in Figure 6. Step-flow growth mode is enhanced by lowering growth pressure. The propagation of BPDs could be promoted under lower growth pressure condition. Thus, the reason that dislocation densities of BPDs and TEDs in epilayers grown at varied pressures are nearly unchanged may be attributed to the contrary effect of increased C/Si ratio and enhanced step-flow growth on the propagation of BPDs. However, further investigations of the relationship between growth pressure and the conversion of BPDs are still required.

**Figure 7:**
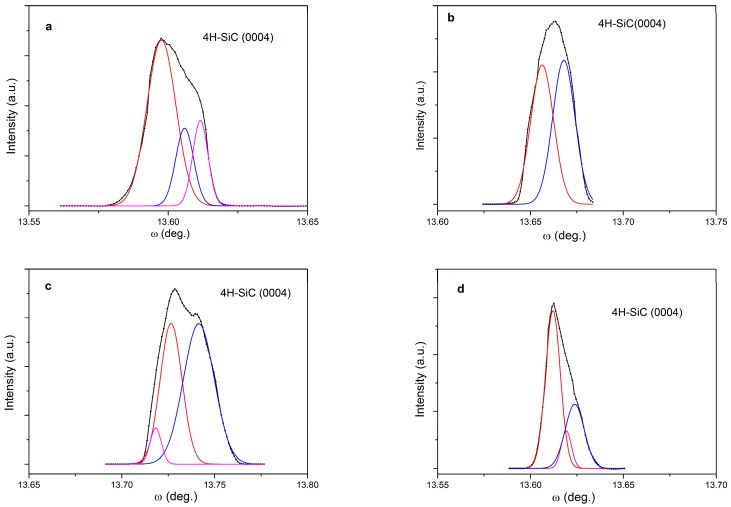
X-ray diffraction (XRD) spectra of epitaxial layers grown at different pressures: (**a**) 100 mbar; (**b**) 80 mbar; (**c**) 60 mbar; (**d**) 40 mbar.

High-resolution X-ray diffraction (HRXRD) rocking curve measurements are employed to study the crystalline quality of the 4H-SiC films performed at different pressures. The reflection peaks from the (0004) plane of 20 μm thick epilayers with full width at half maximum (FWHM) of 50–80 arcsec are shown in Figure 7. It can be seen that the reflection peaks are broadened as more than one domain on samples is probed by the X-ray beam. Therefore, the peaks have been fitted with Gaussians, and each with a FWHM of approximately 20 arcsec, except for the epilayers grown at 80 mbar of which the FWHM is approximately 50 arcsec, as shown in Table 1. This result implies that crystal quality is almost independent of growth pressure. This is consistent with the results shown in Figure 6, since the factors such as crystal defect density and strain could influence FWHM value of diffraction peak theoretically. Compared to the substrate of which the FWHM is more than 40 arcsec, the epilayers with FWHM values of approximately 20 arcsec are of high-quality.

## 3. Materials and Methods

### 3.1. Sample Preparation

Homoepitaxial growth was performed in a commercial Hot-wall chemical vapor deposition (HWCVD) reactor (Epigress VP508, Lund, Sweden) [19,42]. The substrates used for homoepitaxial growth were 1.7 cm × 1.7 cm samples cut from a chemical-mechanical polished n-type, Si-face 4H-SiC wafer oriented 4° off-axis towards the [11,12,13,14,15,16,17,18,19,20] direction with a doping concentration of ~10^18^ cm^−3^. The samples were cleaned following the Radio Corporation of America (RCA) cleaning process prior to deposition. Silane (SiH_4_) and propane (C_3_H_8_) were used as precursors during growth and introduced with a hydrogen carrier gas. In situ etching took place during the temperature ramp from 1400 °C to 1580 °C in pure H_2_ atmosphere with pressure of 100 mbar. Afterwards, the growth pressure was adjusted to a reduced pressure of less than 100 mbar and then the precursors were taken into the chamber. Consequently, the epilayer deposition lasted for a selected period of time. No intentional dopant was introduced during the epitaxial growth. In this paper, the growth temperature and C/Si ratio were maintained at 1580 °C and 1.0 for all the experiments, respectively. The growth pressure was varied between 40 mbar and 100 mbar.

### 3.2. Characterization

The growth rate was calculated from the thickness of the epitaxial layers which was measured by Fourier Transform Infrared Spectroscopy (FTIR) (Spectrum 100, PerkinElmer, Waltham, MA, USA). The epilayer morphology was evaluated with an optical microscope (DM4000M, Leica, Wetzlar, Germany) equipped with Nomarski differential interference contrast optics (NDIC). Atomic force microscopy (Agilent 5500, Agilent, Santa Clara, CA, USA) was used to analyze the surface roughness of the epilayers. The epilayers were etched by molten KOH at 500 °C for 5 min. Dislocation types and densities were also measured using optical microscope. The crystalline quality of the epitaxial layers was studied using X-ray diffraction (XRD) (Bruker D8 Advance, Bruker, Karlsruhe, Germany).

## 4. Conclusions

4H-SiC epilayers were performed in a horizontal hot-wall CVD reactor at low pressure on 4° off-axis substrates. The effects of growth pressure on growth rate, surface roughness and morphological defects were studied. Good stability and reproducibility of the growth processes was verified. At the higher growth pressures of 100 mbar and 80 mbar, the epitaxial films were smooth without step-bunching, but more morphological defects were observed on the films. At lower growth pressures of 60 mbar and 40 mbar, macrosteps formed on the surface increased the surface roughness. However, it can be seen that densities of morphological defects decrease at lower pressure, which indicates that morphological defects tend to appear on the epilayer surfaces without step-bunching rather than on the surfaces with step-bunching. It is suggested that the effect of growth pressure on surface diffusion length is the main reason for the variation of surface morphology. At reduced growth pressure, the increase of surface diffusion length of absorbed adatoms leads to the formation of step-bunching. Meanwhile, morphological defects origining from nucleation on terraces are suppressed as surface diffusion length increases. Besides, the surface energy is mainly released by step-bunching at relatively lower pressure and by morphological defects at relatively higher pressure, respectively. Thus, different dominate modes for minimization of surface energy at varied growth pressure could be another reason for the phenomenon described above. Due to the contrary effect of increased C/Si ratio and enhanced step-flow growth on the propagation of BPDs, the dislocation densities of BPDs and TEDs in epilayers grown at varied pressures are nearly unchanged. High resolution X-ray diffraction measurements indicate that the epilayers performed in this work are of high quality.

## Figures and Tables

**Table 1:** Triangular defect densities and results of Gauss fit of full width at half maximum (FWHM) for epitaxial layers grown at pressure from 40 to 100 mbar.

Growth Pressure (mbar)	100	80	60	40
Densities of TDs (cm^−2^)	80	90	25	7
FWHM (arcsec)	23.544	50.79	25.452	23.256
